# miR-550-1 functions as a tumor suppressor in acute myeloid leukemia via the hippo signaling pathway

**DOI:** 10.7150/ijbs.44365

**Published:** 2020-09-01

**Authors:** Chao Hu, Mengxia Yu, Chenying Li, Yungui Wang, Xia Li, Bryan Ulrich, Rui Su, Lei Dong, Hengyou Weng, Huilin Huang, Xi Jiang, Jianjun Chen, Jie Jin

**Affiliations:** 1 Department of Hematology, the First Affiliated Hospital, College of Medicine, Zhejiang University, 79 Qingchun Road, Hangzhou, 310003, P.R. China.; 2 Department of Cancer Biology, University of Cincinnati, Cincinnati, OH 45219, USA.; 3 Section of Hematology/Oncology, Department of Medicine, University of Chicago, Chicago, Illinois 60637, USA.; 4 Department of Hematology, Affiliated Hangzhou First People's Hospital, Zhejiang University School of Medicine, 216 Huansha Road, Hangzhou, 310006, P.R. China.; 5 Department of Systems Biology & the Gehr Family Center for Leukemia Research, Beckman Research Institute of City of Hope, Monrovia, CA 91016, USA.

**Keywords:** acute myeloid leukemia, miR-550-1, *WWTR1*, apoptosis, proliferation

## Abstract

MicroRNAs (miRNAs) and N^6^-methyladenosine (m^6^A) are known to serve as key regulators of acute myeloid leukemia (AML). Our previous microarray analysis indicated miR-550-1 was significantly downregulated in AML. The specific biological roles of miR-550-1 and its indirect interactions and regulation of m^6^A in AML, however, remain poorly understood. At the present study, we found that miR-550-1 was significantly down-regulated in primary AML samples from human patients, likely owing to hypermethylation of the associated CpG islands. When miR-550-1 expression was induced, it impaired AML cell proliferation both *in vitro* and *in vivo*, thus suppressing tumor development. When ectopically expressed, miR-550-1 drove the G0/1 cell cycle phase arrest, differentiation, and apoptotic death of affected cells. We confirmed mechanistically that WW-domain containing transcription regulator-1 (*WWTR1*) gene was a downstream target of miR-550-1. Moreover, we also identified Wilms tumor 1-associated protein (*WTAP*), a vital component of the m^6^A methyltransferase complex, as a target of miR-550-1. These data indicated that miR-550-1 might mediate a decrease in m^6^A levels via targeting *WTAP*, which led to a further reduction in *WWTR1* stability. Using gain- and loss-of-function approaches, we were able to determine that miR-550-1 disrupted the proliferation and tumorigenesis of AML cells at least in part via the direct targeting of *WWTR1*. Taken together, our results provide direct evidence that miR-550-1 acts as a tumor suppressor in the context of AML pathogenesis, suggesting that efforts to bolster miR-550-1 expression in AML patients may thus be a viable clinical strategy to improve patient outcomes.

## Introduction

Acute myeloid leukemia (AML) is a form of cancer that arises when hematopoietic stem cells (HSCs) undergo oncogenic mutations. In the United States, 19,940 new AML cases are expected to be diagnosed, and 11,180 AML-associated deaths are expected to occur in 2020 (https://www.seer.cancer.gov/). While there have been countless efforts to develop novel therapeutic strategies suited to the treatment of AML, the majority of patients still suffer from poor outcomes, with recent reports estimating a 5-year survival rate of 40% among AML patients^1^, ^2^. As such, there is a clear need to better understand the molecular basis for AML in order to expedite the development of more efficacious therapeutic interventions.

MicroRNAs (miRNAs) are small 20-24 nucleotide non-coding RNA molecules that exhibit endogenous biological functionality via targeting specific downstream mRNAs 3. These miRNAs mediate their activities through their interactions with the RNA-induced silencing complex (RISC), pairing with compatible bases in the 3' untranslated region (UTR) of target mRNAs. These interactions can result in either a suppression of mRNA translation, or a reduction in mRNA stability that lead to mRNA degradation, thus resulting in a marked reduction in target gene expression at the protein level 3, 4. There is now countless evidence that specific miRNAs serve essential regulatory roles in both the context of normal physiology and disease pathogenesis, including leukemogenesis 5-8. Importantly, improved understanding of these miRNAs has led to their utilization for the treatment of certain disorders 9. In hepatitis C mouse models, knockdown of miR-122 led to reduced liver damage and viral load via owing to its ability to regulate several targets, such as mannan binding lectin serine protease 1 (*MASP1*) and prolyl 4-hydroxylase subunit α1 (*P4HA1*) 10. Using locked nucleic acids (LNAs)-modified antimiR-122, preclinical studies were performed in an effort to treat hepatitis C infection. The results indicated this LNAs could bring about a significant reduced liver injure and reduction in infection burden 10. In non-small cell lung cancer (NSCLC), Wiggins JF *et al.* 11 chemically synthesized miR-34a and a lipid-based transport vehicle, and found that this combination effectively blocked cell proliferation by targeting cyclin-dependent kinase 4 (*CDK4*) *in vitro* and *in vivo*. In our previous research, we have identified a set of miRNAs with specific regulatory roles in the context of the proliferation, differentiation, and apoptosis of AML cells. These miRNAs include miR-9, miR-22, miR-26a, miR-150, miR-495, miR-181, miR-126, miR-196b and the miR-17-92 cluster 12-21. In light of our research into miR-150, we ultimately developed a novel FLT3 ligand-binding (amidoamine)-miR-150 nanoparticle (G7-FLT3L-miR-150 nanoparticle) 22, which specifically delivered miR-150 to FLT3-overexpressing AML cells by employing FLT3L as a guiding molecule. Through inhibiting the activation of PIM, AKT, ERK, and STAT5, this nanoparticle displayed a strong anti-leukemic effect *in vitro* and *in vivo*. Even so, however, the specific role of these miRNAs in AML is not completely understood.

WW-domain containing transcription regulator-1 (*WWTR1*), also known as the transcriptional co-activator with PDZ-binding motif (*TAZ*), was first detected based on its status as a 14-3-3 interacting protein 23. *WWTR1* and the paralogous Yes-associated protein (*YAP*) serve as central downstream regulatory factors in the Hippo signaling pathway, which modulates a wide range of cellular processes pertaining to cellular energy status, hypoxia, osmotic stress, tissue organ size, regeneration, homeostasis, and tumorigenesis 24, 25. Indeed, elevated WWRT1 mRNA and protein expression have been found to be associated with the development of gastric, colorectal, breast, and lung cancers 26-30. Consistent with this, elevated WWTR1 protein level has been determined to be a risk factor for the development of both glioblastoma multiforme and colorectal cancer 31, 32. In one study, Justice *et al.* 33 also determined that there was an association in gastric cancer between increased WWTR1 expression and tumor TNM stage as well as incidence of lymph node metastasis. Wang *et al.*^27^ further found that WWTR1 expression in NSCLC led to its regulation of Cyclin A and C transforming growth factor (CTGF), which in turn led to a disruption of apoptosis in neoplastic cells. Recent work has revealed that large tumor suppressor (LATS)-mediated YAP/WWTR1 phosphorylation was the key regulatory event controlling the activity of these proteins in cells 34. Consistent with this, Jimenez-Velasco *et al.* 35 found *LATS1* and *LATS2* to be downregulated in leukemia as a consequence of their hypermethylation, and reduced *LATS2* expression has been found to be associated with worse outcomes among leukemia patients. This suggests the possibility that a reduction in LATS1/2 activity may underlie the alterations in YAP/WWTR1 stabilization and activation in the context of leukemia. However, clarity is still needed regarding the mechanisms governing increased WWTR1 activity in AML.

In the present study, we for the first time provided evidence that miR-550-1 was significantly downregulated in AML. Moreover, when overexpressed, miR-550-1 was able to impair AML cell proliferation and oncogenesis both *in vitro* and *in vivo* owing to its ability to regulate the Hippo signaling pathway. We further found *WWTR1* to be a direct miR-550-1 target, thereby at least partially explaining its role in regulating AML progression.

## Materials and Methods

### Cell line and patients' samples

We cultured MV4-11 and Kasumi-1 cells in RPMI-1640 (Invitrogen, Carlsbad, USA) containing 10% heat-inactivated FBS (Gibco, Grand Island, USA), 1% penicillin-streptomycin (Sigma-Aldrich, St Louis, USA) and 1% HEPES (Sigma-Aldrich). Murine progenitor cells were cultured in RPMI 1640 containing 10 ng/ml interleukin 3 (IL-3) (Peprotech, Rocky Hill, USA), 10 ng/ml IL-6 (Peprotech), 100 ng/ml stem cell factor (SCF) (Peprotech), 55 nM 2-mercaptoethanol (BME) (Sigma-Aldrich), 1% HEPES, 10% FBS, and 1% PS. The AML patients' samples were acquired from the First Affiliated Hospital of Zhejiang University and the University of Chicago Hospital with informed consent. The study was approved by the institutional review board of two hospital's ethics committee.

### Measurement of cell viability

A MTT assay (Promega, Madison, USA) was used to measure viability based on provided directions. Briefly, MV4-11 and Kasumi-1 cells were plated into 96-well plates (10000 cells/100 μL), with dye solution added to wells at the indicated time points. After 4-hour 37°C incubation, stop buffers were added and cell absorbance was assessed the following day at 570 nm.

### Flow cytometry

A BD LSRII Flow Cytometer was used in all analyses, and FlowJo v10 was used for data analysis. For measurements of apoptosis, 0.5×10^6^ cells were stained with an Annexin V-APC Apoptosis Detection Kit (BD Biosciences, San Diego, USA) based on provided directions.

For cell cycle analyses, 0.5×10^6^ cells were fixed overnight at 4°C in 75% ethanol, washed thrice in PBS, and stained using propidium iodide for 20 minutes.

For immunophenotyping analyses, BM, PB, and spleen cells (0.5×10^6^) were collected, washed thrice using PBS, and stained at 4°C with antibodies specific for CD11b, CD117, CD45.1, CD45.2, and Gr-1 (BD Biosciences) for 20 minutes. After two additional washed, cells were then fixed in a fixation buffer prior to analysis.

### Western blotting

A total of 5×10^6^ cells were washed in cold PBS prior to lyse with RIPA buffer (Pierce, Rockford, USA) supplemented with PMSF, EDTA, and protease inhibitors. Samples underwent 30 min of centrifugation at 12000×g, after which supernatants were isolated and loaded in equal protein amounts (30-50 ug) onto gels for SDS-PAGE analysis. After separation, proteins were transferred onto PVDF membranes which were then blocked with 5% skim milk in TBST for 1 h, followed by probing overnight at 4°C with anti-β-ACTIN (#3700), anti-WWTR1 (#83669), anti-PARP (#9532), anti-AKT (#4685), anti-p-AKT (#4060), anti-CDK6 (#13331), anti-Rb (#9309), anti-p-Rb (#8516), anti-E2F1 (#3742), anti-CCND1 (#2978), anti-BCL-2 (#15071), anti-p27 (#3686) (Cell Signal Technology, Beverly, USA), or anti-c-myc (Abcam, Cambridge, USA, ab32072). A peroxidase-conjugated secondary antibody was then applied to blot for 1 h, followed for four washed with TBST, after which chemi-luminescence (ECL, Pierce) was used to detect protein bands.

### Quantitative RT-PCR

A miRNeasy kit (Qiagen, Frederick, USA) was used for extracting total RNA from 1×10^6^ cells based on provided directions. cDNA was then synthesized from 1 µg of this RNA via M-MLV reverse transcriptase (Invitrogen, Carlsbad, CA, USA). A 7900HT real-time PCR system (Applied Biosystems, Foster City, USA) was employed for qPCR analyses, with SYBR Green used to setup triplicate reactions assessing relative mRNA expression. For miRNA expression, TaqMan qPCR was conducted according to provided directions (Applied Biosystems). The 2^-ΔΔCt^ method was used to calculate miRNA and mRNA relative expression, which was normalized to endogenous levels of *U6* and *GAPDH*, respectively.

### Plasmid and virus production

The pri-miR-550-1 sequence was amplified by PCR from healthy human BM mononuclear cells (MNCs). Primers with mutated sequences (Table 1) were then used to generate the indicated mutant miR-550-1 template, and these wild type (WT) and mutant miR-550-1 sequences were thereafter cloned into the MSCV-PIG vector (MSCV-Puromycin-IRES-GFP vector) (Cold Spring Harbour Laboratory, USA) in order to overexpress these two miRNA isoforms. These sequences were inserted between the XhoI (CTCGAG) and EcoRI (GAATTC) sites in this vector. For *WWTR1*-CDS vectors, the WT sequence was amplified from healthy human BM MNCs prior to insertion into the pCDH vector (SBI, Mountain View, USA). The MSCVneo-*MLL-AF9* plasmid was kindly provided by Dr. Scott Armstrong.

One day prior to transfection, 5 ×10^5^ HEK293T cells were plated into 60-mm dishes. Retroviruses were then produced via transfecting cells with vector DNA and a packaging vector (PCL-Eco or PCL-Ampho) with the Effectene Transfection Kit (Qiagen). The *WWTR1* overexpressing lentivirus was generated via co-transfection of the *WWTR1*-pCDH plasmid and packaging lentivirus vectors (pRSV-Rev, pMDLg/pRRE and pMD2.G). At 48 and 72 h post-transfection, cellular supernatants were harvested and filtered through a 0.45 μm cellulose acetate filter prior to storage.

### Target gene analysis

miRNA target gene predictions were made through the use of the PITA (http://genie.weizmann.ac.il/pubs/mir07/), miRBase Targets (http://microrna.sanger.ac.uk), TargetScan (http://www.targetscan.org), and miRanda (http://www.microrna.org) miRNA-target gene prediction databases.

### Dual luciferase reporter and mutagenesis assay

We conducted dual luciferase reporter and mutagenesis assays based on a modified version of a previously reported protocol 16. The *WWTR1* 3'-UTR sequences containing putative miR-550-1-binding sites were synthesized via PCR utilizing the following primers: forward 5'-GGGCACTAGTATTCGACCTGATTTACAGTTTC-3' and reverse 5'- TATTACGCGTTGAGATCAGGAGTTTGAGAAC-3'. The resultant fragment underwent insertion into the pMIR-REPORT vector (Ambion, Austin, USA). In addition, a mutated version of this 3'-UTR fragment was generated using primers bearing the mutant sequence. A total of 6,000 HEK293T cells were plated per well of a 24-well plate in triplicate, and following overnight culture these cells were co-transfected with pMIR-REPORT-*WWTR1* or mutant pMIR-REPORT-*WWTR1* vectors and MSCV-PIG-miR-550-1, mutant MSCV-PIG-miR-550-1, or MSCV-PIG empty vectors (20ng each). The β-galactosidase vector (1ng) (Ambion) was additionally transfected into all experimental cells, and after a 48 h incubation all cells were lysed. Relative luciferase activity was then measured via a Dual-Light Combined Reporter Gene Assay System (Applied Biosystems).

### Colony forming/replating assay

Colony formation assays were performed in accordance to a modified version of a previously reported protocol 16. Donor murine BM cells were isolated from 6-week old B6.SJL (CD45.1) after injecting 5-fluorouracil (5-FU; 150mg/kg) for 5 days, and hematopoietic progenitor cells were isolated via a Mouse Lineage Cell Depletion Kit (Miltenyi Biotec Inc., Auburn, USA). These progenitor cells then underwent infection with the indicated viruses using a spinoculation protocol with the assistance of polybrene 36-38. Following transduction, cells were incubated overnight at 37^o^C in fresh media, and this was then repeated the following day. Thereafter, 2 × 10^4^ cells were plated in methylcellulose medium (Stem Cell Technologies Inc, Vancouver, Canada) containing 10 ng/ml IL-3, IL-6, granulocyte-macrophage colony-stimulating factor (GM-CSF), 30 ng/ml SCF, and 2 μg/ml puromycin and/or 1 mg/ml G418, as appropriate. After incubating for 7 days, colony formation in each of the experimental groups was assessed, and these colonies were then replated.

### Primary and secondary bone marrow transplantations (BMT)

For BMT assays, donor mice were B6.SJL (CD45.1+), and recipient mice were C57BL/6 (CD45.2+). All animal experiments were approved by the local Institutional Animal Care and Use Committee (IACUC).

In the primary BMT assays, BM cells from healthy donor mice (B6.SJL) were transduced with the indicated retrovirus combinations (MSCV-PIG + MSCVneo-*MLL-AF9*, MSCV-PIG-miR-550-1 + MSCVneo-*MLL-AF9,* and mutant MSCV-PIG-miR-550-1 + MSCVneo-*MLL-AF9*). The resultant donor cells were then mixed with helper cells (BM cells from a healthy C57BL/6 mouse) at a ratio of 3 × 10^5^ to 1 × 10^6^ per recipient mouse. These cells were then injected into the tail vein of an 8-week old lethally irradiated (960 rads) recipient mouse. In secondary BMT assays, these lethally irradiated recipient mice were injected using leukemic BM cells that had been isolated from the initial primary recipient mice, and no helper cells were added.

Once recipient mice exhibited signs of systemic illness, peripheral blood (PB) samples were collected via tail bleeding in order to establish whole blood counts. Engraftment was evaluated via flow cytometry based on CD45.1 expression in PB samples. Moribund mice were euthanized, and liver, thymus, and spleen weight was determined. BM cells were collected from euthanized animals and prepared for cytospin slides, which then underwent Wright-Giemsa staining.

### m^6^A dot blot assay

We isolated total RNA with the miRNeasy kit and quantified RNA levels. Next, RNA samples were spotted using a Bio-Dot Apparatus (Bio-Rad, Hercules, USA) on Amersham Hybond-N+ membranes (GE Healthcare, Chicago, USA), and a UV cross-linker was then used to cross-link them to this membrane. Membranes then underwent two washes using Milli-Q, followed by treatment for 10 min using 0.02% methylene blue (Sigma-Aldrich). Membranes were then rinsed until the dye was washed away from background regions, and dots of methylene blue were then imaged. Next, 5% nonfat dry milk was used for membrane blocking for 1 h, after which an antibody against m^6^A (1:2000 dilution, Synaptic Systems, Goettingen, Germany, #202003) was used to probe blots at 4°C overnight. Membranes were then washed thrice in TBST and probed at room temperature using HRP-conjugated goat anti-rabbit IgG for 1 h, prior to visualization with an ECL system.

### Statistical analysis

SPSS v16 (IBM, Armonk, USA) was used to compare all experimental results via Student's t-tests or two-way ANOVAs. Data are given as means ± standard deviations (SDs) from at least three repeat experiments. The Kaplan-Meier approach was used to assess overall survival. *P*<0.05 was the threshold of statistical significance.

## Results

### miR-550-1 is down-regulation in AML

**Figure 1:**
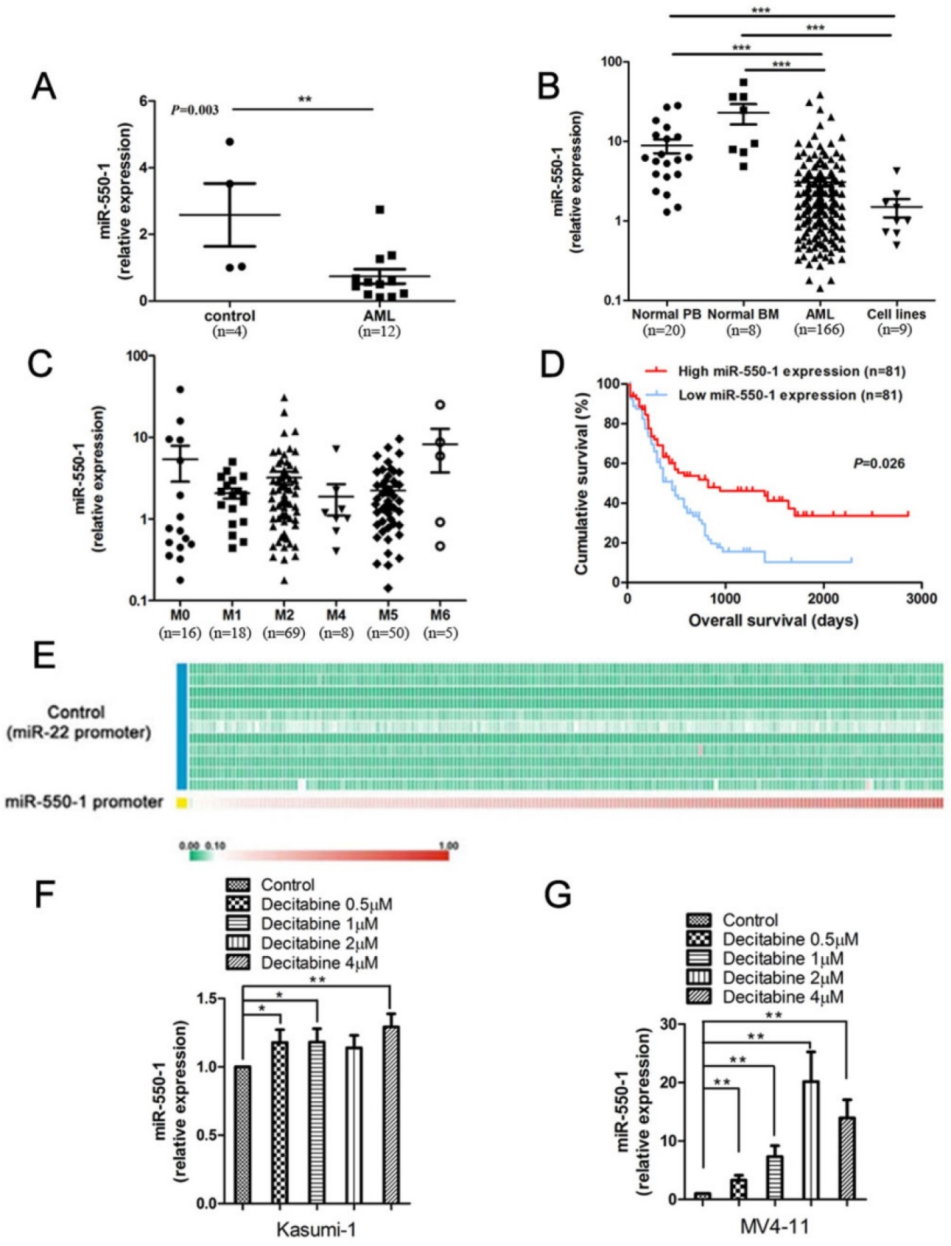
Human AML samples exhibit significantly decreased miR-550-1 expression and altered miR-550-1 DNA methylation. (**A**) Taqman qPCR was used to assess miR-550-1 expression in MNC samples from 12 AML patients and 4 normal controls (NC). (**B**) Taqman qPCR was used to assess miR-550-1 expression in MNC samples from 166 AML patients, as well as in 8 normal bone marrow (BM) samples, 20 samples of normal peripheral blood (PB), as well as 9 leukemic cell lines. (**C**) Relative miR-550-1 expression was assessed in various AML subtypes. (**D**) Reduced miR-550-1 expression levels were found to correlate with poorer overall survival in the TCGA dataset; (**E**) The pri-miR-550-1 promoter CpG islanded exhibited hypermethylation, unlike those of pri-miR-22 in 194 AML patient samples from the TCGA_194S dataset; (**F, G**) We treated the Kasumi-1 and MV4-11 cells using a range of DNA methylation inhibitor concentrations (0, 1, 2, or 4 µmol decitabine), revealing decitabine treatment to increased miR-550-1 expression relative to controls. Error bar indicates SD of triplicate experiments *, *P*<0.05; **, *P*<0.01; ***, *P*<0.001.

We have previously detected a set of specific miRNAs that were down-regulated in AML samples with t(8; 21), inv(16), t(15; 17), or mixed lineage leukemia (MLL) rearrangements, relative to normal controls (NC) 21. Of these previously identified miRNAs, miR-550-1 was among the most significantly down-regulated in the AML cohort. We therefore first confirmed that miR-550-1 was significantly down-regulated in AML patients using bone marrow (BM) mononuclear cell (MNC) samples from 12 patients with primary AML (from University of Chicago Hospitals), revealing a marked decrease in the expression of this miRNA as compared to NC samples (n=4) (*P*=0.003) (Fig. 1A). We then extended these findings to a larger 166 patient primary AML sample cohort (from the First Affiliated Hospital of Zhejiang University) (Table 2 and Table S1), using qPCR to confirm that miR-550-1 was significantly down-regulated in AML patients relative to NC samples, which included 8 normal BM samples and 20 normal peripheral blood (PB) samples (*P*<0.001) (Fig. 1B). Similarly, miR-550-1 expression was markedly decreased in 9 leukemic cell lines (including MV4-11, Kasumi-1, MOLM-13, MONOMAC-6, OCI-AML3, NB4, THP-1, HL-60, OCI-AML2) relative to controls (*P*<0.001) (Fig. 1B). In addition, the expression level of miR-550-1 was significantly increased at complete remission (CR) compared to initial diagnosis (*P*=0.010) (Figure S1). We did not find any significant differences with respect to miR-550-1 expression as a function of the French-American-British (FAB) subtype (Fig. 1C). Given its low expression in AML patients, we next sought to determine whether the level of miR-550-1 expression correlated with patient clinical outcomes. Using a 162 patient TCGA AML dataset, we divided patients based on whether they expressed high or low levels of miR-550-1, as determined based on the median expression level of this miRNA. In so doing we found that patients with lower miR-550-1 expression exhibited poorer overall survival (OS) than did those with higher expression (*P*=0.026; Fig. 1D). Together, these findings validated our previous results with respect to patterns of miR-550-1 in AML, and strongly suggested the possibility that this miRNA may serve as a tumor suppressive role in the context of leukemogenesis.

Promoter methylation is known to be a key regulator of many miRNAs in the context of AML, including miR-126 and miR-375 13, 39. Whereas we have previously found the miR-22 promoter to be hypomethylated 15, when we assessed this TCGA dataset we found that the pri-miR-550-1 promoter region was hypermethylated (Fig. 1E). Consistent with these findings, when the MV4-11 and Kasumi-1 AML cell lines were treated using the hypomethylating agent, decitabine, miR-550-1 expression rose significantly (Fig. 1F, G). In addition, the methylation degree of miR-550-1 CpG islands was apparently inhibited with decitabine (Figure S2). These findings are therefore consistent with a model wherein miR-550-1 promoter hypermethylation in AML cells may lead to its reduced expression.

### miR-550-1 suppresses tumor development *in vitro*

**Figure 2:**
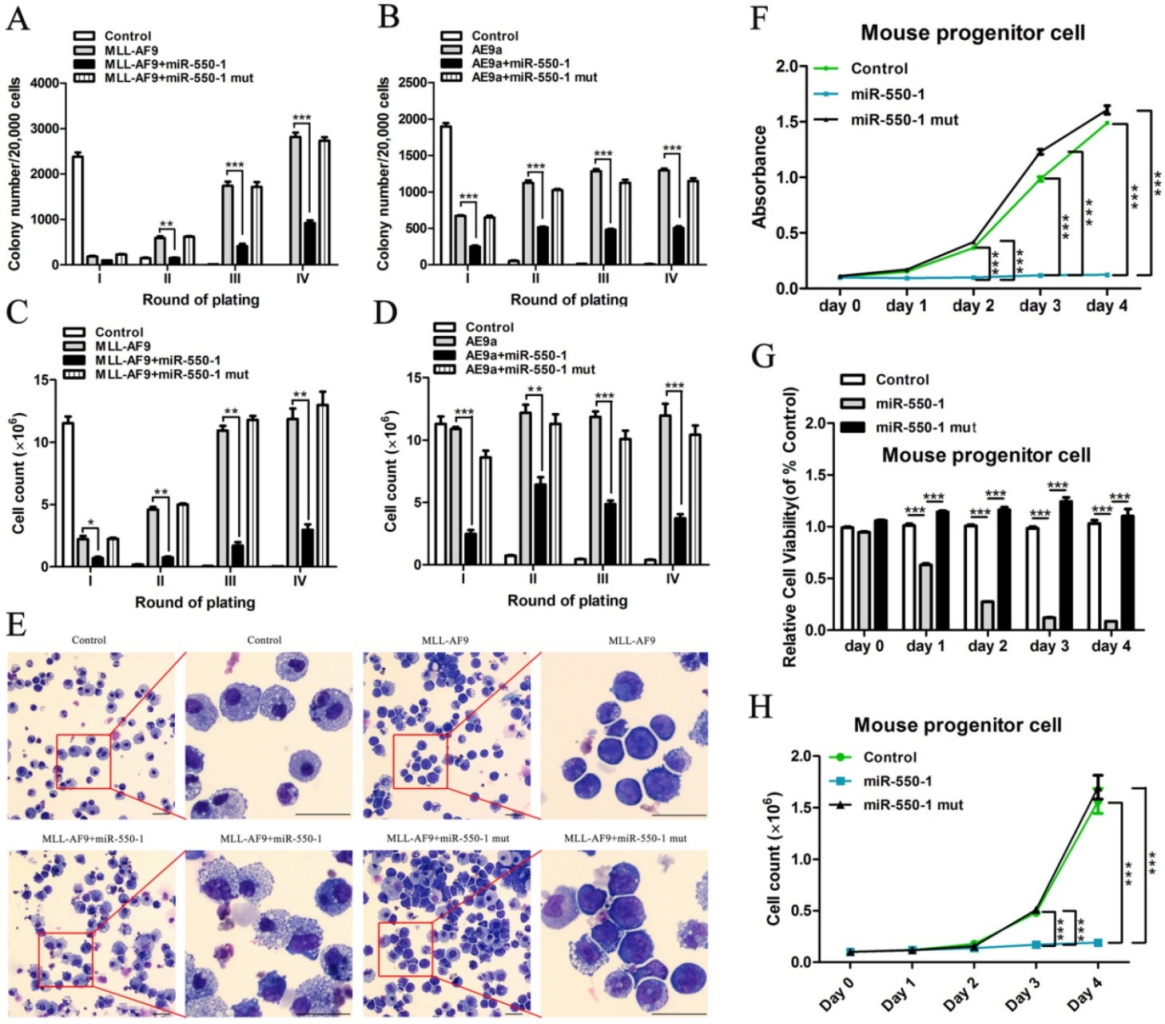
miR-550-1 suppresses tumor cell growth *in vitro.* (**A-D**) *MLL-AF9* or *AE9a* -induced colony formation following replating was dramatically reduced upon over-expression of wild-type but not mutant miR-550-1. (**E**) Forced ectopic expression of wild-type miR-550-1, but not of mutant miR-550-1, led to a significant enhancement of cell differentiation as determined via cytospin analyses. (**F-H**) Ectopic forced expression of wild-type but not mutant miR-550-1 led to a reduction in the viability and proliferation of murine progenitor cells. Error bar indicates SD of triplicate experiments. *, *P*<0.05; **, *P*<0.01; ***, *P*<0.001. Scale bar = 10 µm.

To explore the biological role of miR-550-1 in AML cells, we first conducted colony formation assays (CFA). To this end, MSCVneo-*MLL-AF9 +* MSCV-PIG (as control) 40, MSCVneo-*MLL-AF9* + MSCV-PIG-miR-550-1 (i.e., MLL-AF9 + miR-550-1), MSCVneo-*MLL-AF9* + MSCV-PIG-miR-550-1 mutant (i.e., MLL-AF9 + miR-550-1 mut), MSCVneo-*AE9a* (*AML1-ETO9a* fusion gene; a truncated *AML1-ETO* fusion gene causing rapid AML onset in mice 41) *+* MSCV-PIG (as control), MSCVneo-*AE9a* + MSCV-PIG-miR-550-1 (i.e., AE9a + miR-550-1) or MSCVneo-*AE9a* + MSCV-PIG-miR-550-1 mutant (i.e., AE9a + miR-550-1 mut) were separately co-transduced into normal murine BM progenitor cells prior to replating on methylcellulose medium. Following a 7 day incubation period, the media was then replaced and equivalent culture conditions were maintained for each group. We observed a marked reduction in colony formation capabilities for BM progenitor cells induced with the MLL-AF9 or AE9a fusion proteins following induction of miR-550-1 expression (Fig. 2A-D). Indeed, such ectopic miR-550-1 expression led to a clear reduction in the viability and proliferation of MLL-AF9 fusion protein-induced murine BM progenitor cells (Fig. 2E-H). When the 6-base miR-550-1 seed sequence was mutated, this led to a complete ablation of the miR-550-1-dependent suppression of the proliferation of these AML-like cells (Fig. 2A-H), suggesting that miR-550-1 exerts these observed effects via a canonical base-pairing mechanism regulating target gene expression. Notably, when miR-550-1 was overexpressed, this led to a marked induction of cell differentiation (Fig. 2E).

**Figure 3:**
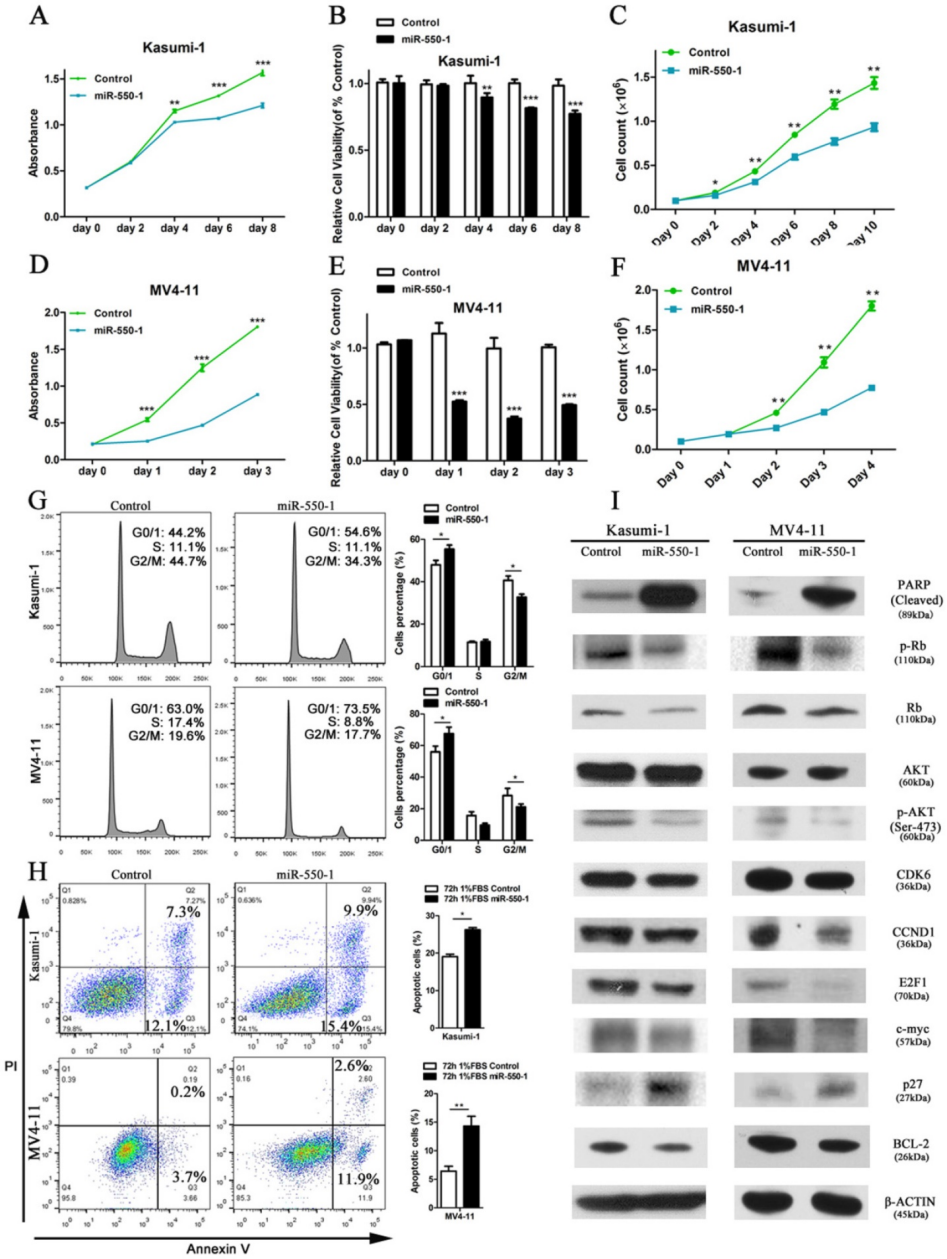
Ectopic miR-550-1 expression reduced the viability and proliferation of Kasumi-1 and MV4-11 cells. (**A, B**) Using MTT assays revealed that ectopic miR-550-1 expression led to a marked reduction in the viability of Kasumi-1 cells beginning on day 4. (**C**) The inhibitory effects of ectopic miR-550-1 expression on Kasumi-1 cells were assessed. (**D, E**) Using MTT assays revealed that ectopic miR-550-1 expression led to a marked reduction in the viability of MV4-11 cells beginning on day 1. (**F**) The inhibitory effects of ectopic miR-550-1 expression on MV4-11 cells were assessed. (**G**) Ectopic miR-550-1 expression led to the induction of G0/1 phase arrest in both Kasumi-1 and MV4-11 cells. (**H**) miR-550-1 overexpression led to the apoptotic death of both Kasumi-1 and MV4-11 cells; (**I**) Western blotting assessment of proteins related to apoptosis, proliferation, and the cell cycle in Kasumi-1 and MV4-11 cells with or without elevated miR-550-1 expression. Error bar indicates SD of triplicate experiments. *, *P*<0.05; **, *P*<0.01; ***, *P*<0.001.

In order to assess the role of miR-550-1 in the context of AML biology, we next used the MV4-11 and Kasumi-1 human AML cell lines to conduct gain-of-function experiments. We found that forced ectopic miR-550-1 expression led to a clear reduction in the viability and proliferation for both of these cell lines (Fig. 3A-F). Furthermore, miR-550-1 overexpression led to a marked increase in the apoptotic cell death and G0/1 phase arrest (Fig. 3 G and H). Consistent with these results 42, 43, western blotting confirmed that the levels of the G0/1-S checkpoint regulatory molecules CCND1 and CDK2 were reduced upon ectopic miR-550-1 expression, as well as the levels of the cell cycle regulatory proteins p-Rb, E2F1, and P27. We also observed clear decreases in the levels of the proliferation-associated proteins p-AKT and c-myc, as well as the anti-apoptotic BCL-2 protein upon ectopic miR-550-1 expression, whereas levels of the pro-apoptotic PARP protein were markedly increased in these same cells (Fig. 3I). Together these results strongly suggest that miR-550-1 significantly reduces rates of AML cell proliferation and leukemic cell transformation proliferation *in vitro*.

### miR-550-1 inhibits AML leukemogenesis *in vivo*

**Figure 4:**
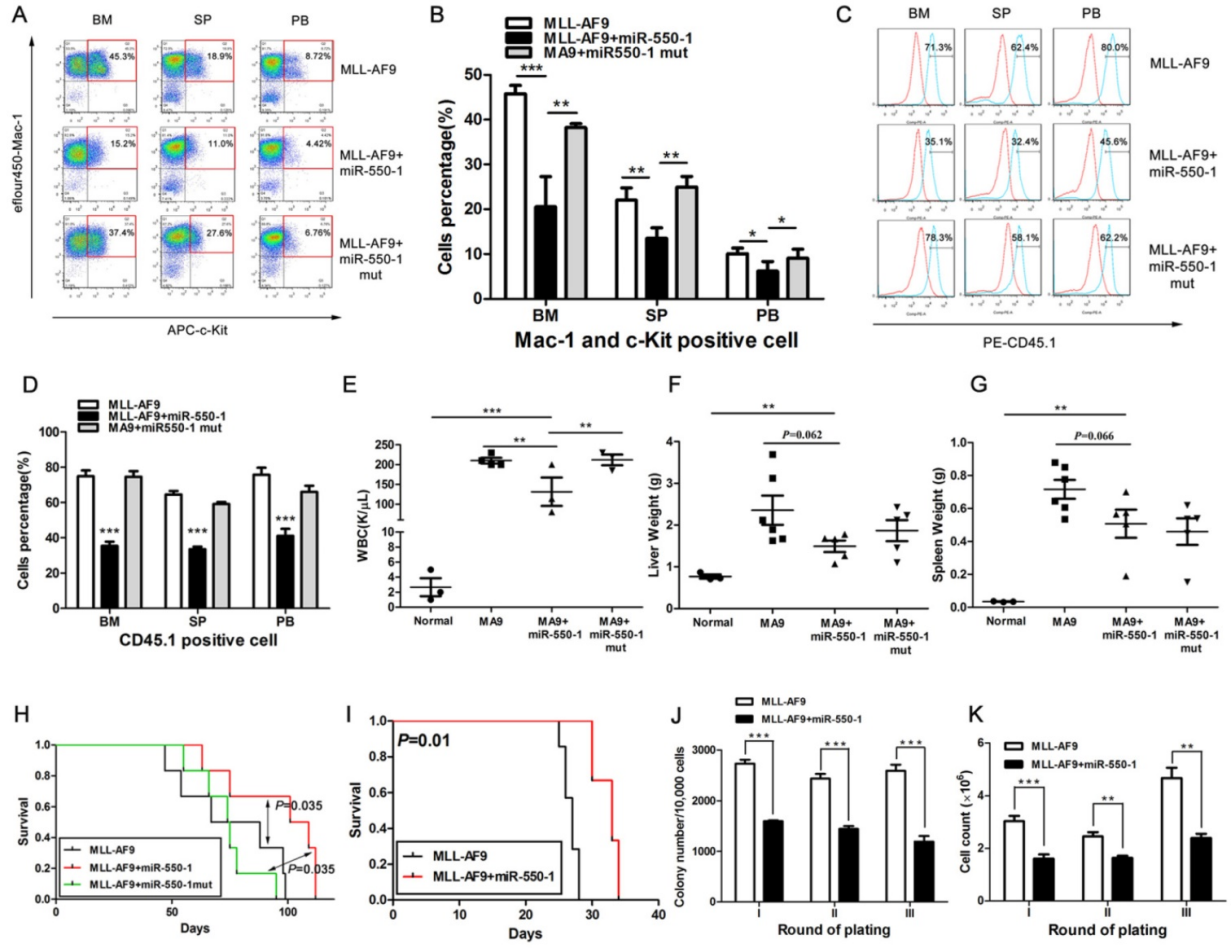
miR-550-1 is able to disrupt MLL-fusion-mediated leukemogenesis *in vivo*. (**A, B**) Overexpression of wild-type but not mutant miR-550-1 reduced the proportion of c-Kit^+^ blast cells in the BM, SP and PB. (**C, D**) Induced expression of wild-type, but not mutant miR-550-1, led to a clear reduction of the engraftment frequency in the BM, SP and PB samples. Red line: isotype control, blue line: CD45.1. (**E-G**) miR-550-1 overexpression led to a clear reduction in PB, liver, and splenic tumor burden. (**H**) Animals in the MLL-AF9+miR-550-1 group had a markedly slower rate of leukemia development than did those in the primary BMT MLL-AF9 only group (*P*=0.035). (**I**) Leukemic development was markedly reduced for the secondary BMT MLL-AF9+miR-550-1 group relative to the MLL-AF9 only group (*P*=0.010); (**J and K**) Overexpression of wild-type but not mutant miR-550-1 led to a marked reduction in the colony formation capabilities of secondary BMT leukemic cells. Error bar indicates SD of triplicate experiments. *, *P*<0.05; **, *P*<0.01; ***, *P*<0.001.

We next explored whether miR-550-1 was able to suppress the development of leukemia *in vivo* as it did *in vitro* by using a primary BMT assay system. Briefly, we co-transduced murine BM progenitor cells (B6.SJL, CD45.1) with the MSCVneo-*MLL-AF9* + MSCV-PIG (as control), MSCVneo-*MLL-AF9* + MSCV-PIG-miR-550-1 (i.e., MLL-AF9 + miR-550-1), or MSCVneo-*MLL-AF9* + MSCV-PIG- miR-550-1 mutant (i.e., MLL-AF9 + miR-550-1 mut) vectors. Transduced cells were then injected into the tail veins of recipient mice (C57BL/6, CD45.2). By flow cytometry, we found all mice in the MLL-AF9+miR-550-1 group displayed an apparent decline in the proportion of c-Kit^+^ blast cells in the BM, spleen (SP), and PB compared to control or MLL-AF9+miR-550-1 mutant group (Fig. 4A and B). Moreover, we then assessed engraftment, revealing that forced ectopic miR-550-1 expression was associated with a clear reduction in engraftment rates and tumor burdens in the BM, PB, liver, and spleen (Fig. 4C-G). Importantly, mice in the MLL-AF9 + miR-550-1 group exhibited a significantly longer latency for AML development as compared with either the control group or the MLL-AF9 + miR-550-1 mutant group (both *P*<0.05; Fig.4H). Together, these findings strongly suggest that inducing the expression of miR-550-1 in these cells leads to a significant reduction in the induction of primary leukemogenesis by the *MLL-AF9* fusion gene.

We additionally utilized secondary BMT assays as a means of assessing the importance of miR-550-1 in the maintenance of AML following its development. We found that mice in the miR-550-1 + MLL-AF9 group exhibited slower AML development than did those in the MLL-AF9 only group (median overall survival: 33 vs. 27 days, *P*=0.010; Fig. 4I), suggesting that suppressing miR-550-1 did contribute to the sustained maintenance of AML driven by the *MLL-AF9* fusion gene. Secondary CFA further provided confirmation that miR-550-1 overexpression was linked to delayed leukemogenesis (Fig. 4J and K). Our results together therefore provide clear evidence for the role of miR-550-1 as a tumor suppressor in the context of AML development and maintenance.

### Identification of potential miR-550-1 target genes

**Figure 5:**
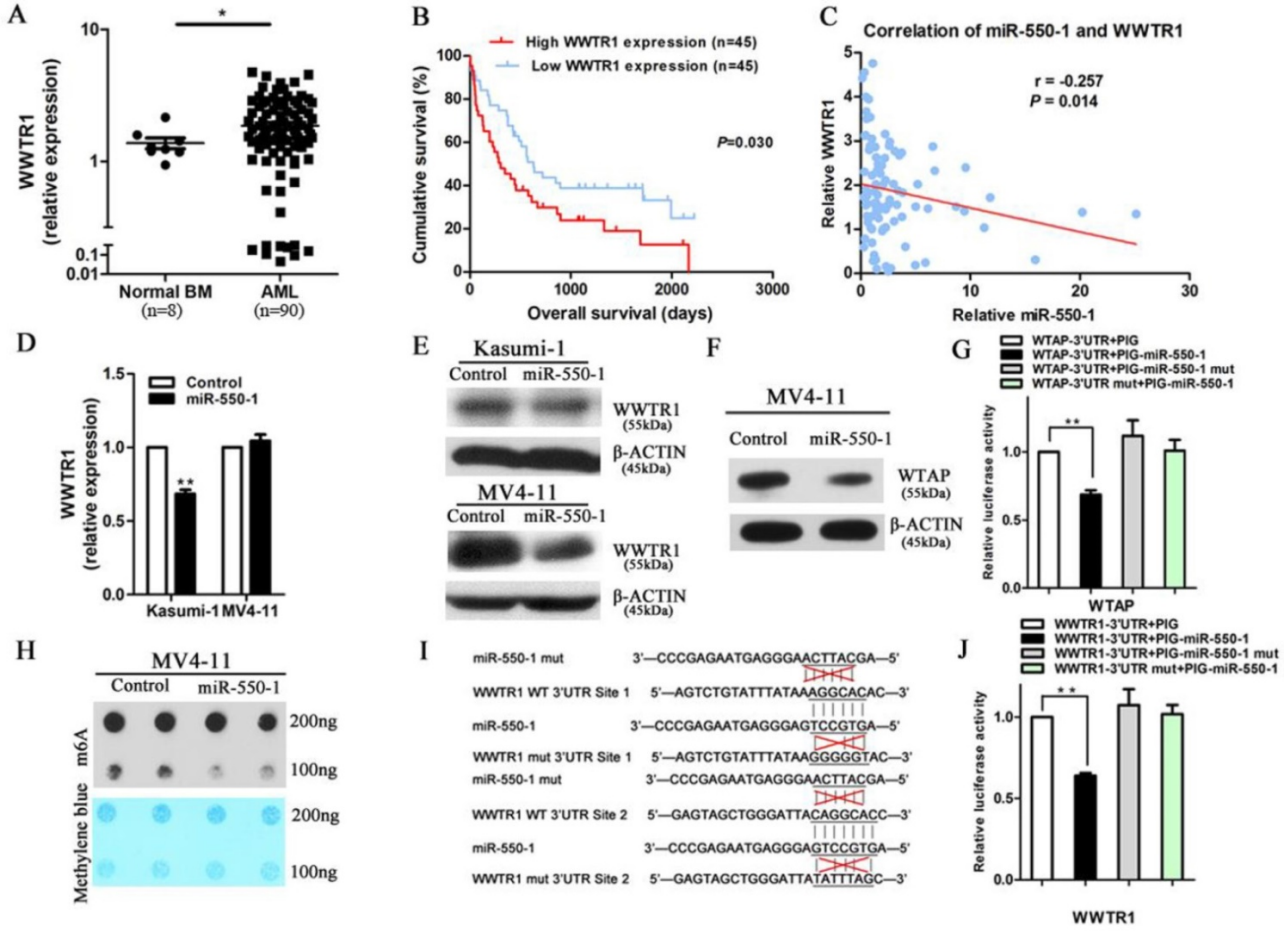
*WWTR1* is a direct miR-550-1 target. (**A**) qPCR-mediated assessment of *WWTR1* expression in AML and NC samples. (**B**) Lower *WWTR1* expression was associated with improved overall survival in AML patients relative to higher expression (*P*=0.030). (**C**) There was a significant negative correlation between miR-550-1 and *WWTR1* expression (r=-0.257, *P*=0.014). (**D, E**) Western blotting and qPCR confirmed the ability of miR-550-1 to inhibit endogenous *WWTR1* expression. (**F**) Western blotting suggested that *WTAP* may be a miR-550-1 target. (**G**) miR-550-1 was able to significantly suppress luciferase activity for a vector bearing the *WTAP* 3'-UTR without affecting a control vector. (**H**) A m^6^A dot blot analysis in MV4-11 cells that were or were not overexpressing miR-550-1. (**I**) miR-550-1 target sites in the *WWTR1* 3'-UTR were mutated. (**J**) miR-550-1 led to a significant suppression of luciferase activity for a vector containing the *WWTR1* 3'-UTR without affecting a control vector. Error bar indicates SD of triplicate experiments. *, *P*<0.05; **, *P*<0.01.

miRNAs are able to mediate their biological activities through the suppression of specific target genes. Given that our results suggested that miR-550-1 played a central role in suppressing the development of leukemia through regulating the cell cycle and inducing apoptosis, we next sought to identify potential miR-550-1 target genes linked to the promotion of apoptosis and G0/1 phase arrest. We therefore employed the TargetScan, PITA, miRanda, and miRBase programs in order to predict potential targets, identifying a total of 4,941 putative target genes identified by a minimum of 1 of these programs. Of the genes identified via this approach, *WWTR1* was of potential interest as it scored highly among the predicted genes. *WWTR1* has previously been found to serve as a key transcriptional co-activator in the Hippo signaling pathway, and there were multiple reports indicating that it could positively regulate both the cell cycle (specifically the G1/S transition) and mitochondrially-induced apoptosis in a range of cancer types 26, 28, 44. We therefore first sought to assess *WWTR1* mRNA expression in AML patient samples, revealing it to be significantly upregulation in these patients' samples relative to NC samples (*P*<0.05; Fig. 5A). Kaplan-Meier survival curves also confirmed that higher *WWRT1* mRNA levels were correlated to poorer overall survival outcomes in these patients (*P*=0.030; Fig. 5B). This suggests that *WWTR1* may serve as an oncogenic function in AML, and may also be a miR-550-1 target gene.

Using the TCGA and CALGB datasets, we performed *in silico* analyses revealing a negative correlation between miR-550-1 and *WWTR1* expression (r=-0.173, *P*=0.021; r=-0.291, *P*=0.007, respectively). We additionally examined the expression of miR-550-1 and *WWTR1* in 90 AML samples in our cohort, again revealing a significantly negative correlation between these two factors (r=-0.257, *P*=0.014; Fig. 5C). We also confirmed that the expression of WWTR1 was reduced at both the mRNA and protein level upon miR-550-1 overexpression in AML cells (Fig. 5D and E). Curiously, in the MV4-11 cells, the WWTR1 was downregulated only at the protein but not at the mRNA level upon miR-550-1 overexpression, suggesting that miR-550-1 might primarily affect the *WWTR1* mRNA stability. Previous work suggested that Wilms tumor 1-associated protein (*WTAP*) targeting could lead to alterations in mRNA stability as a function of m^6^A modification 45, 46. Our results also suggested that *WTAP* was a direct miR-550-1 target gene (Fig. 5F and G). Induced ectopic expression of miR-550-1 also led to a reduction in global mRNA m^6^A levels (Fig. 5H), potentially contributing at least in part to the reduced *WWTR1* stability observed in our results.

To further confirm that there was a direct interaction between miR-550-1 and *WWTR1*, we next utilized a luciferase reporter assay system, generating luciferase reporter constructs bearing either a WT or mutated form of the *WWTR1* 3'-UTR in the pMIR-REPORT vector (Fig. 5I). When utilized in cells, we found that miR-550-1 was able to significantly decrease the luciferase activity associated with promoters bearing the WT *WWTR1* 3'-UTR (*P*=0.002), whereas they had no effect on those bearing the mutated *WWTR1* 3'-UTR (Fig. 5J). These results thus provide strong evidence supporting the fact that *WWTR1* is a miR-550-1 target.

### *WWTR1* is a key miR-550-1 target in AML

**Figure 6:**
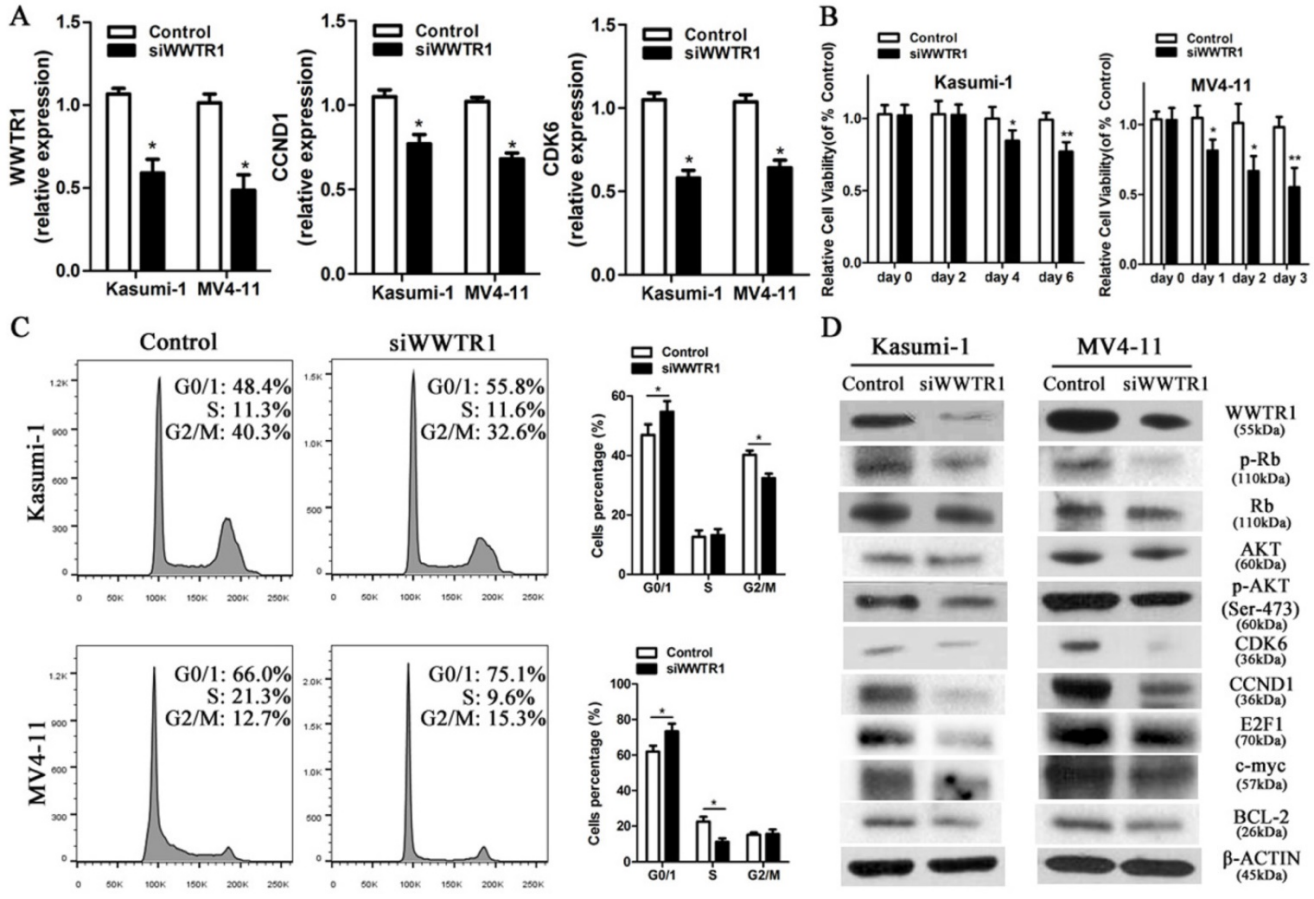
Silencing *WWTR1* partly recapitulates miR-550-1-associated phenotypes. (**A**) qPCR-mediated assessment of *WWTR1*, *CCND1,* and *CDK6* expression in Kasumi-1 and MV4-11 cells following siWWTR1 transfection. (**B**) MTT-mediated assessment revealing that *WWTR1* silencing led to a reduction in Kasumi-1 and MV4-11 cell viability. (**C**) Silencing of *WWTR1* resulted in G0/1 phase arrest in both Kasumi-1 and MV4-11 cells. (**D**) Western blotting assessment of proteins related to apoptosis, proliferation, and the cell cycle in Kasumi-1 and MV4-11 cells with or without decreased WWTR1 expression. Error bar indicates SD of triplicate experiments. *, *P*<0.05; **, *P*<0.01.

**Figure 7:**
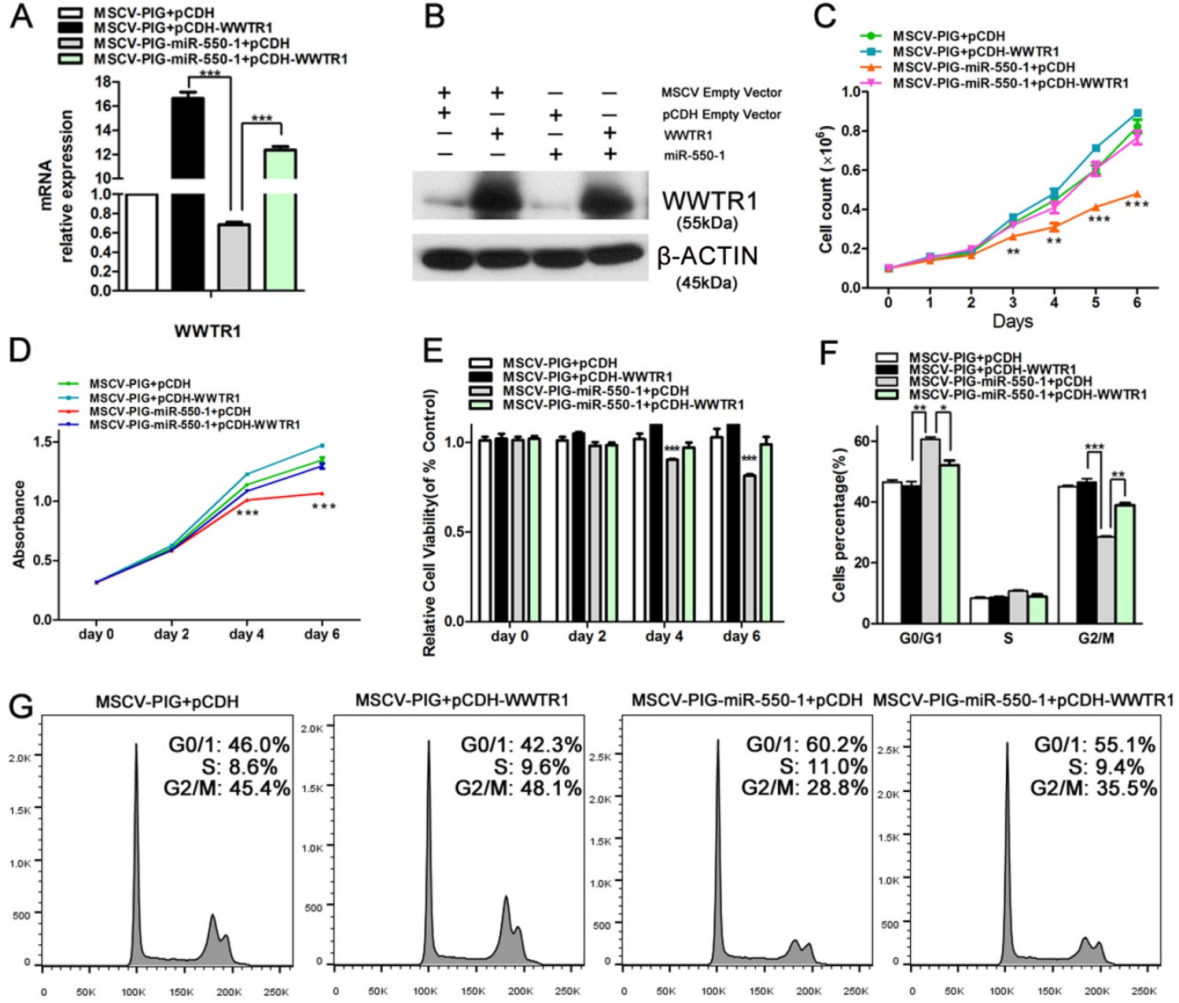
Inducing *WWTR1* expression can partially rescue miR-550-1-associated phenotypes. (**A, B**) Elevated ectopic WWTR1 expression was confirmed in miR-550-1-transfected or control Kasumi-1 cells by qPCR and Western blotting. (**C**) miR-550-1-mediated inhibition of cell proliferation was partially rescued via overexpressing *WWTR1* in Kasumi-1 cells beginning on day 3. (**D, E**) MTT-mediated assessments revealed that elevated ectopic *WWTR1* expression partly overcame miR-550-1-dependent reductions in cell viability beginning on day 4. (**F, G**) Elevated ectopic *WWTR1* expression led to a partial rescue of miR-550-1-dependent G0/1 phase arrest. Error bar indicates SD of triplicate experiments. *, *P*<0.05; **, *P*<0.01; ***, *P*<0.001.

As *WWTR1* is known to have important roles in the context of cancer 26, 47, 48, we next performed loss- and gain-of-function experiments in order to ascertain as to whether *WWTR1* contributed to the anti-leukemic activity of miR-550-1. At present, the role of *WWTR1* in AML has not been specifically assessed. We therefore employed a small interfering RNA construct to silence WWTR1 expression at the mRNA and protein level (Fig. 6A and D), and this led to a marked decrease in the proliferation of Kasumi-1 and MV4-11 cells, which instead underwent G0/1 arrest (Fig. 6B and C). We found that WWTR1 knockdown was linked to decreased protein levels of CCND1, CDK6, p-Rb, E2F1, BCL-2, p-AKT, and c-myc (Fig. 6D), phenocopying the anti-leukemic activity of miR-550-1. When WWTR1 was instead overexpressed in Kasumi-1 cells, this led to an increase in cell viability and proliferation, largely reversing the inhibitory effects of miR-550-1 (Fig. 7A-E). When WWTR1 and miR-550-1 were co-expressed, this led to a reversal in miR-550-1-mediated suppression of cell proliferation and G0/1-phase arrest in Kasumi-1 cells (Fig. 7F and G). Together, these findings thus suggest that miR-550-1-mediated suppression of WWTR1 expression at least partially governs the anti-leukemic activity of this miRNA.

## Discussion

Herein, we produce evidence that miR-550-1 plays a role in suppressing the development of AML and the proliferation of AML cells, instead promoting their apoptotic death. In humans, miR-550-1 is encoded in the 7p14.3 locus, which is a non-coding section of the *ZNRF2* gene. Landgraf *et al.* first identified this miRNA via small RNA library sequencing in 2007, but it has not previously been studied in depth in the context of cancer 49. To our knowledge, no previous reports have shown that miR-550-1 plays a vital role in leukemogenesis. We determined that miR-550-1 expression was decreased in two independent primary AML patient cohorts, and elevated miR-550-1 expression was associated with higher hemoglobin (Hb) levels (Table 2), potentially contributing to improved patient prognosis. However, at present there is no strong evidence regarding a link between miR-550-1 and Hb, and as such further research is required. Consistent with our findings regarding the methylation of the miR-550-1 region, we found that decitabine was able to partially reverse miR-550-1 downregulation, suggesting methylation of this region in AML patients contributed to miR-550-1 dysregulation.

In order to explore the specific mechanisms governing the link between miR-550-1 and reduced leukemia severity, we first conducted an *in silico* target analysis approach. We then, using luciferase reporter and mutagenesis assays, confirmed that *WWTR1*, which is an oncogene in solid tumors 28, 30, 31, was one key miR-550-1 target. Noguchi *et al.* 28 recently found that elevated *WWTR1* expression was associated with poorer overall survival in those with NSCLC, and that it was confirmed to be an independent inferior element in NSCLC patients (HR=1.34, 95%CI 1.01-1.76, *P*=0.040). Similarly, breast cancer patients with elevated *WWTR1* expression also exhibit poorer outcomes 30. Furthermore, there is a positive correlation between *WWTR1* expression and poorer prognosis, increased tumor invasion, and metastasis in those with gastric cardia adenocarcinoma 50. Consistent with these past results, we observed significant upregulation of *WWTR1* mRNA expression in AML, and this expression was negatively correlated with that of miR-550-1. *YAP* and *TAZ* have been reported to be transcriptional coactivators capable of recognizing cognate cis-regulatory elements via interactions with additional transcription factors, such as TEA domain family members (*TEAD*) 51, 52. Li *et al.* 26 and Noguchi *et al.* 28 found WWTR1 to be capable of regulating the cell-cycle and apoptosis via regulating CCND1, CCND3, c-myc, BCL-2, and DKK1 expression, with WWTR1 knockdown resulting in a G1/S transition block in the cell cycle. YAP and TAZ have also been found to upregulate BCL-2-family member transcription, thereby suppressing the mitochondrial pathway of apoptosis 44. Consistent with this, we found that both miR-550-1 overexpression and WWTR1 knockdown reduced CCND1, CDK6, p-Rb, E2F1, BCL-2, and p-AKT levels, and increased p27, and PARP levels. When ectopic *WWTR1* lacking the 3'-UTR region was overexpressed, this significantly rescued the miR-550-1-induced G0/1-phase arrest observed *in vitro*. To date, the function of *WWTR1* in AML was not previously determined, but our study thus highlights for the first time that *WWTR1* is a key mediator related to the anti-leukemic activity of miR-550-1.

m^6^A is the most common methylation event modifying mRNA molecules in mammals, regulating a range of processes such as heat shock, differentiation, DNA damage responses, tissue development, and miRNA processing 53-55. There is clear evidence that a disruption in m^6^A is linked to the pathogenesis of AML 56, 57. WTAP is a m^6^A methyltransferase complex component, regulating m^6^A methyltransferase activity. Recent work indicates that WTAP is able to improve CDK2 stability via binding to the 3'UTR region, thereby enhancing cell proliferation in renal carcinoma 45. Bansal *et al.* 58 found that WTAP knockdown reduced the proliferation of AML cells, suggesting it serves as an oncogenic function. Our work highlights, for the first time, that miR-550-1 mediates its anti-leukemic activity at least in part via decreasing *WWTR1* stability by targeting *WTAP*.

Although we found that impairing *WWTR1* mRNA stability, rather than promoting its degradation, was the primarily regulatory role of miR-550-1 in MV4-11 cells, further clarification is needed to determine why this effect was not identical in both cell lines. Whether miR-550-1/YAP/WWTR1 interact in a manner so as to form a negative feedback loop in AML remains unknown. Interestingly, a report by Chaulk *et al.* 59 suggested that nuclear YAP and WWTR1 together induced DICER complex activity, thus suggesting that these proteins might promote the maturation of certain pre-miRNAs into their mature forms in certain contexts. Liu *et al.* 60 also found circRNA-000425 to be a YAP/WWTR1 target using a circRNA microarray analysis, and revealed that YAP/WWTR1 were able to promote the oncogenic activity of miR-17 and miR-106b via inhibiting circRNA-000425 transcription. Additional experiments, however, are needed to determine whether YAP and WWTR1 regulate miR-550-1 expression. Our findings indicate that miR-550-1 is able to inhibit cell proliferation and promote apoptosis of AML cells via, at least in part, vying the direct targeting of the *WWTR1* 3'-UTR. Ultimately our findings both identify a novel tumor-suppressor miRNA, and also characterize previously unknown regulatory pathways governing WWTR1 expression in AML.

In summary, our study reveals the following: (1) elevated miR-550-1 expression is a favorable prognostic indicator in AML, and in AML patients it is at least partially dysregulated due to the hypermethylation promoter; (2) miR-550-1 is able to promote apoptosis and inhibit proliferation via regulation of the WTAP/WWTR1/BCL-2 and WTAP/WWTR1/CDK6/Rb/E2F1 pathways in AML; (3) m^6^A modifications are important for regulating the ability of miR-550-1 to target WWTR1. Our results thus demonstrate that miR-550-1 is a latent factor which suppresses AML, and as such enhancing expression of this miRNA may be a valuable therapeutic strategy in those with AML.

## Supplementary Material

Supplementary figures and tables.

## Figures and Tables

**Table 1:** Oligonucleotide sequences

Name	Sequence (5'->3')
miR-550-1 precursor F	CTGGTGCAGTGCCTGAGGGAGTAAG
miR-550-1 precursor R	CTTACTCCCTCAGGCACTGCACCAG
mutant miR-550-1 F	CTGGTGCAGCATTCAAGGGAGTAAG
mutant miR-550-1 R	CTTACTCCCTTGAATGCTGCACCAG
WWTR1-3'UTR F	GGGCACTAGTATTCGACCTGATTTACAGTTTC
WWTR1-3'UTR R	TATTACGCGTTGAGATCAGGAGTTTGAGAAC
WWTR1-qPCR F	GATCCTGCCGGAGTCTTTCTT
WWTR1-qPCR R	CACGTCGTAGGACTGCTGG
WWTR1-CDS-F	TTCTATCTAGAATGAATCCGGCCTCGG
WWTR1-CDS-R	TTTTGCGGCCGCTTACAGCCAGGTTAGAA

F: forward primer; R: reverse primer.

**Table 2:** High and low miR-550-1 expressing AML patient characteristics

	miR-550-1		*P* value
	Low expression	High expression	
Number	83	83	
**Age**			0.740
<60	55	57	
≥60	28	26	
**Gender**			0.431
Male	46	51	
Female	37	32	
**2016 WHO classification**			0.732
AML with recurrent genetic abnormalities			
AML with mutated *NPM1*	23	25	
AML with biallelic mutations of *CEBPA*	10	10	
AML not otherwise specified			
AML with minimal differentiation	8	6	
AML without maturation	4	3	
AML with maturation	18	19	
Acute myelomonocytic leukemia	5	1	
Acute monoblastic/monocytic leukemia	15	18	
Pure erythroid leukemia	0	1	
Hb median (range) (g/L)	81.0 (51.0-136.0)	88.4 (50.0-135.0)	0.041
PLT median (range) (×109/L)	46.5 (20.4-110.1)	51.2 (22.1-109.2)	0.580
WBC median (range) (×109/L)	12.5 (0.7-236.9)	12.4 (0.2-222.5)	0.936
BM blasts median (range) %	72.5 (28.4-90.5)	70.2 (25.2-82.3)	0.449
**Genes mutations, n (%)**			
*NPM1*	23 (27.7)	25 (30.1)	0.732
*CEBPA^DM^*	10 (12.4)	10 (12.4)	1.000
*FLT3-ITD*	23 (27.7)	18 (21.7)	0.368
*DNMT3a*	12 (14.5)	7 (8.4)	0.223
*IDH1*	5 (6.0)	12 (14.5)	0.073
*IDH2*	14 (16.9)	6 (7.2)	0.056

WHO: World Health Organization; Hb: hemoglobin; PLT: platelet; WBC: white blood cell; BM: bone marrow; DM: double-allele mutation. Patients were separated into groups based on their miR-550-1 expression level relative to the median value, with 83 each in the high and low expression groups.

## Abbreviations

AML: acute myeloid leukemia
miRNAs: microRNAs
HSC: hematopoietic stem cells
WWTR1: WW-domain containing transcription regulator-1
TAZ: transcriptional co-activator with PDZ-binding motif
YAP: yes-associated protein
CTGF: C transforming growth factor
LATS: large tumor suppressor
qRT-PCR: quantitative RT-PCR
BM: bone marrow
MNC: mononuclear cells
CFA: colony forming/replating assays
ALL: acute lymphoblastic leukemia
