# Supplementary material for: miR-550-1 functions as a tumor suppressor in acute myeloid leukemia via the hippo signaling pathway
Source: Int J Biol Sci. 2020 Sep 1;16(15):2853–67. doi: 10.7150/ijbs.44365 (PMC7545716; doi:10.7150/ijbs.44365)
Supplement: Supplementary file 1 — Supplementary figures and tables. [file ijbsv16p2853s1.pdf]

Supplementary information

miR-550-1 Functions as A Tumor Suppressor in Acute Myeloid Leukemia via The Hippo Signaling Pathway

Chao Hu[1, 2, 3*]; Mengxia Yu[4*]; Chenying Li[1, 2*]; Yungui Wang[1, 2, 3*]; Xia Li[1]; Bryan Ulrich[3]; Rui Su[2, 5]; Lei Dong[2, 5]; Hengyou Weng[2, 5]; Huilin Huang[2, 5]; Xi Jiang[2, 3, 5†]; Jianjun Chen[2, 3, 5†]; Jie Jin[1†]

[1]Department of Hematology, the First Affiliated Hospital of Zhejiang University, Hangzhou, 310003, PR China
[2]Department of Cancer Biology, University of Cincinnati, Cincinnati, OH 45219, USA
[3]Section of Hematology/Oncology, Department of Medicine, University of Chicago, Chicago, Illinois 60637, USA
[4]Department of Hematology, Hangzhou First People's Hospital, College of Medicine, Zhejiang University, Hangzhou, 310006, PR China
[5]Department of Systems Biology & the Gehr Family Center for Leukemia Research, Beckman Research Institute of City of Hope, Monrovia, CA 91016, USA
*Chao Hu, Mengxia Yu, Chenying Li and Yungui Wang contributed equally to this work.

†Correspondence: Jie Jin, Department of Hematology, The First Affiliated Hospital of Zhejiang University, Hangzhou, Zhejiang 310003, China; email: jiej0503@zju.edu.cn; Jianjun Chen, Department of Systems Biology & the Gehr Family Center for Leukemia Research, Beckman Research Institute of City of Hope, Monrovia, CA 91016, USA; email: jianchen@coh.org; or Xi Jiang, Department of Systems Biology & the Gehr Family Center for Leukemia Research, Beckman Research Institute of City of Hope, Monrovia, CA 91016, USA; email: xjiang@coh.org.

Supplementary Figure

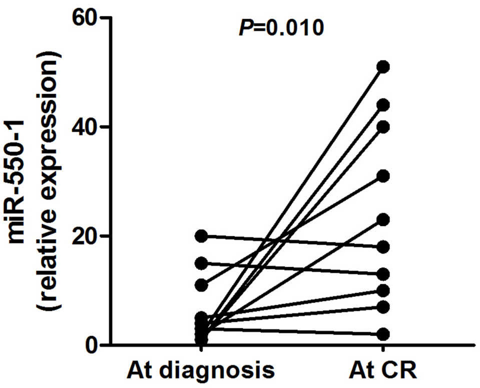

Supplementary Figure S1. The expression level of miR-550-1 was significantly increased at complete remission compared to initial diagnosis.

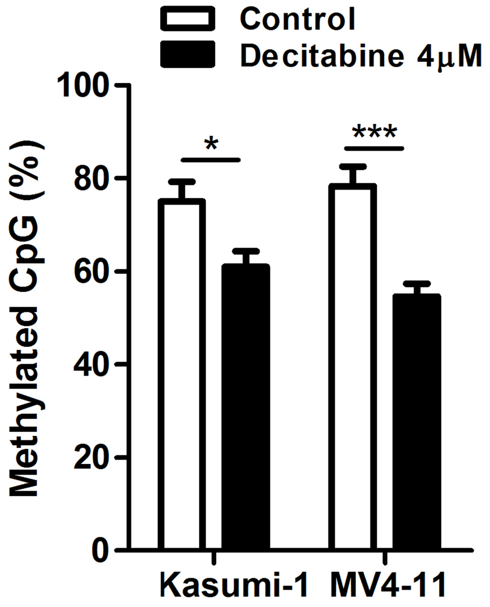

Supplementary Figure S2. Decitabine apparently reduced the methylation level of miR-550-1 CpG islands in Kasumi-1 and MV4-11 cell lines. Error bar indicates SD of triplicate experiments. *, P<0.05; ***, P<0.001.

Supplementary Tables

Table S1. The FAB High and low miR-550-1 expressing AML patient characteristics.

	miR-550-1 low expression	miR-550-1 high expression	*P* value
Number	83	83	
FAB subtype			0.601
M0	10	6	
M1	9	9	
M2	33	36	
M4	6	2	
M5	23	27	
M6	2	3	

FAB: French-American-British.

Patients were separated into groups based on their miR-550-1 expression level relative to the median value, with 83 each in the high and low expression groups.
